# The rs225017 Polymorphism in the 3′UTR of the Human *DIO2* Gene Is Associated with Increased Insulin Resistance

**DOI:** 10.1371/journal.pone.0103960

**Published:** 2014-08-08

**Authors:** Leonardo B. Leiria, José M. Dora, Simone M. Wajner, Aline A. F. Estivalet, Daisy Crispim, Ana Luiza Maia

**Affiliations:** Thyroid Section, Endocrine Division, Hospital de Clínicas de Porto Alegre, Universidade Federal do Rio Grande do Sul, Porto Alegre, RS, Brazil; Central China Normal University, China

## Abstract

The Thr92Ala (rs225014) polymorphism in the type 2 deiodinase (*DIO2*) gene has been associated with insulin resistance (IR) and decreased enzyme activity in human tissues but kinetic studies failed to detect changes in the mutant enzyme, suggesting that this variant might be a marker of abnormal *DIO2* expression. Thus, we aimed to investigate whether other *DIO2* polymorphisms, individually or in combination with the Thr92Ala, may contribute to IR. The entire coding-region of *DIO2* gene was sequenced in 12 patients with type 2 diabetes mellitus (T2DM). Potentially informative variants were evaluated in 1077 T2DM patients and 516 nondiabetic subjects. IR was evaluated using the homeostasis model assessment (HOMA-IR) index. *DIO2* gene sequencing revealed no new mutation but 5 previously described single nucleotide polymorphisms (SNPs). We observed that all T2DM patients displaying high HOMA-IR index (n = 6) were homozygous for the rs225017 (T/A) polymorphism. Further analysis showed that the median fasting plasma insulin and HOMA-IR of T2DM patients carrying the T/T genotype were higher than in patients carrying the A allele (P = 0.013 and P = 0.002, respectively). These associations were magnified in the presence of the Ala92Ala genotype of the Thr92Ala polymorphism. Moreover, the rs225017 and the Thr92Ala polymorphisms were in partial linkage disequilibrium (|D′| = 0.811; *r* ^2^ = 0.365). In conclusion, the rs225017 polymorphism is associated with greater IR in T2DM and it seems to interact with the Thr92Ala polymorphism in the modulation of IR.

## Introduction

Thyroid hormones are critical to the development, growth and metabolism of virtually all tissues [1], [2]. The iodothyronine deiodinases types 1 (D1), 2 (D2) and 3 (D3) are selenoenzymes of the oxidoreductase family which catalyze iodine removal from the outer (D1 and D2) or inner (D1 and D3) ring of thyroid hormones. While D1 and D2 convert T4 to the metabolically active hormone T3, D3 inactivates both T4 and T3 [2]. In humans, D2 is the most important deiodinase for intracellular T3 generation in target tissues [1], [2] and, together with D1, contributes for 80% of peripheral T3 [3]. The gene that encodes D2 (*DIO2*) is expressed in the thyroid, pituitary, brain, heart, placenta, skeletal muscle and adipocytes [4], [5], [6], [7], [8], [9]. D2 activity is tightly regulated at transcriptional, post-transcriptional and post-translational levels [10], [11], which thereby supports the hypothesis that this enzyme plays an important homeostatic role in metabolism.

Type 2 diabetes mellitus (T2DM) is a heterogeneous group of disorders usually characterized by varying degrees of insulin insufficiency and insulin resistance (IR), which result in increased blood glucose concentrations. Ultimately, IR results either from inappropriately increased hepatic gluconeogenesis or decreased glucose disposal rate in tissues such as skeletal muscle, adipose tissue and liver [12].

Glucose homeostasis may be affected by thyroid status [13], [14]. Thyroid hormone affects insulin action in skeletal muscle and adipose tissue in part by upregulating the expression of the glucose transporter 4 (GLUT4) and, therefore, increasing glucose uptake [15], [16]. An increase in both hepatic endogenous glucose disposal and insulin mediated-glucose uptake is observed in patients with hyperthyroidism when compared with euthyroid subjects [17], [18], [19]. In contrast, studies showed that, in hypothyroidism, there is a decrease in both glucose disposal [19] and insulin mediated-glucose uptake in muscle, and also an impaired ability of insulin to stimulate glucose disposal related to insulinaemia [19], [20]. In animal models, experimental induction or spontaneous forms of thyroid dysfunction are also associated with impaired glucose tolerance [20]. Mice with targeted disruption of the *dio2* gene have higher insulin levels during glucose tolerance tests and reduced glucose uptake during insulin tolerance tests, consistent with the occurrence of IR [21]. According to these findings, it is plausible to postulate that a lower intracellular D2-generated T3 in skeletal muscle might create a state of relative intracellular hypothyroidism, decreasing the expression of genes involved in energy use, such as GLUT4, and thus resulting in increased IR.

Previous studies have demonstrated that polymorphisms in the *DIO2* gene might interfere in the phenotypic expression of D2 [7], [22]. The *DIO2* single-nucleotide polymorphism (SNP), in which a threonine (Thr) changes to alanine (Ala) at codon 92 (Thr92Ala, rs225014, A/G), was associated with IR in obese white women and with a 20% lower glucose disposal rate in white non-diabetic women [23]. The Ala92Ala genotype was also associated with a more pronounced IR in patients with T2DM and the frequency of this variant allele was found to be increased in some ethnic groups, such as Pima Indians and Mexican-Americans, who have a higher prevalence of IR [23]. Even though some studies have failed to demonstrate an association between the Thr92Ala polymorphism and glycemic traits or T2DM [24], [25], [26], the association between this polymorphism and T2DM is supported by data reported in a recent systematic review and meta-analysis [27]. However, the mechanism by which this occurs is still not clear. Decreased D2 activity has been observed in human tissue biopsy samples [7], [27] but studies failed to identify any changes in the *ex vivo* biochemical properties of the mutant enzyme [7], [28], suggesting that this variant might be only a marker for abnormal *DIO2* expression. Here, we aimed to determine whether other SNPs in the *DIO2* gene, individually or in combination with the Thr92Ala polymorphism, contribute to the IR phenotype.

## Materials and Methods

### Ethics statement

The information obtained during the study did not affect the patients' diagnosis or treatment. The protocol was approved by the Committee on Research Ethics from Hospital de Clínicas de Porto Alegre, and all subjects signed an informed consent term. Clinical investigation was conducted according to the principles expressed in the Declaration of Helsinki.

### Study population

In the first phase of this study, we used direct automated sequencing of the *DIO2* gene to search for SNPs in 12 T2DM patients with different degrees of IR. These 12 patients were selected from two subgroups in our T2DM population: 6 patients with a low HOMA-IR index (ranging from 0.32 to 1.3) and 6 patients with a high HOMA-IR index (6.2–23.4). The results generated by sequencing the *DIO2* gene in T2DM patients were compared with the sequences of the human *DIO2* gene available in GenBank (http://www.ncbi.nlm.nih.gov/genbank/).

In the second phase of the study, allele and genotype frequencies of a potentially informative *DIO2* variants found by sequencing were compared between 1077 patients with T2DM and 516 nondiabetic subjects. The criteria used to select the variant for further analysis were: 1) the polymorphism was not known to be a neutral variation; 2) it involved change between chemically different amino acids or was localized at regulatory regions, such as the promoter or 3′- untranslated region (3′UTR); 3) its frequency was not low (minor allele frequency less than 5% in the literature or GenBank); and 4) the frequency of the variant was different in the groups under analysis (in this case, low and high HOMA-IR groups) [29], [30]. Additionally, haplotypes constructed from the combination of this polymorphism with the Thr92Ala polymorphism were analyzed regarding their effect on IR in T2DM patients.

The 1077 patients with T2DM were selected from a multicenter study that started recruiting patients in southern Brazil in 2002 and whose purpose was to investigate risk factors for T2DM and its complications. Initially, it included 4 tertiary-teaching hospitals in the Brazilian state of Rio Grande do Sul, namely Grupo Hospitalar Conceição, Hospital São Vicente de Paula, Hospital Universitário de Rio Grande and Hospital de Clínicas de Porto Alegre. The detailed description of that study can be found elsewhere [31]. The group of subjects without diabetes was composed of 516 healthy volunteers who came to Hospital de Clínicas de Porto Alegre to donate blood.

### Clinical and anthropometric profiles and laboratory analyses

A standard questionnaire was used to collect information from all patients about age, age at T2DM diagnosis and drug treatment. All patients with T2DM were white. The ethnic group was defined on the basis of self-classification and subjective classification (skin color, nose and lip shapes, hair texture and family history).

All T2DM patients underwent physical and laboratory evaluations. They were weighed (barefoot and wearing light outdoor clothing) and had their height measured. Body mass index (BMI) was calculated as weight (kg) divided by height squared (m^2^). Blood pressure (BP) was measured twice, in the sitting position, with a 5-min rest between measurements, using a mercury sphygmomanometer (Korotkoff phases I and V). The mean of the two measurements was used to record systolic and diastolic BP. Arterial hypertension was defined as BP≥140/90 mmHg or use of antihypertensive drugs regardless of BP at the time of assessment.

Serum samples for laboratory tests were collected after a 12-h fast from all patients with T2DM. Glucose levels were determined by a glucose oxidase method; creatinine by the Jaffé reaction; glycated haemoglobin (A1C) by an ion-exchange HPLC procedure (Merck-Hitachi L-9100 glycated haemoglobin analyzer, Merck, Darmstadt, Germany; reference range: 2.7–6.0%); and total plasma cholesterol, HDL-cholesterol, and triglycerides, by enzymatic methods. LDL-cholesterol was calculated using the Friedewald equation. Serum insulin was measured by radioassay (Elecsys Systems 1010/2010/modular analytics E170, Roche Diagnostics, Indianapolis, IN). Insulin sensitivity was estimated by the HOMA-IR index [fasting insulin (milliunits per millilitre) x fasting glucose (millimoles per litre)/22.5] [32]. The mean HOMA-IR index of control subjects in our laboratory was 1.84±1.02 [33].

### Molecular analysis

DNA was extracted from peripheral blood leukocytes by a standardized salting-out procedure. A search for variants of the *DIO2* gene was performed in 12 patients with T2DM by direct sequencing of all exons, partial intron sequences (500 bp of exon-intron junctions), and 5′UTR and 3′UTR sequences (1000 bp each, including the selenocysteine insertion sequence element - ESECIS - in the 3′UTR). Screening of this limited number of individuals may fail to detect some rarer polymorphisms; however, this number seems to be adequate to identify representative variants which are sufficiently polymorphic to warrant association studies. Direct sequencing in an automated ABI 3100 *Avant Genetic Analyzer* (Life Technologies, Foster City, CA, USA) was performed using ABI Prism Big Dye Terminator Cycle Sequence Ready reaction kit (Life Technologies) according to the manufacturers' recommendations, and using primers described in **Table S1**.

Potentially informative variants identified through the sequencing of the *DIO2* gene were selected for subsequent genotyping in all patients with T2DM and nondiabetic subjects. These SNPs, together with the rs225014 (Thr92Ala) polymorphism, were determined using primers and probes contained in Human Custom TaqMan Genotyping Assays 40x (Assays-By-Design Service, Life Technologies). Sequences of primers and probes used for genotyping are shown in **Table S1**. Reactions were conducted in 96-well plates, at a total reaction volume of 5 µL and using 2 ng of genomic DNA, TaqMan Genotyping Master Mix 1x (Life Technologies), and Custom TaqMan Genotyping Assay 1x. Plates were then placed in a real-time PCR thermal cycler (7500 Fast Real-Time PCR System, Life Technologies) and heated for 10 minutes at 95°C, followed by 45 cycles of 95°C for 15 seconds and 62°C for 1 minute. Fluorescence data files of each plate were analyzed using automated allele-calling software (SDS 2.1, Life Technologies).

### Statistical analyses

Results are expressed as mean ± SD, % or median (minimum-maximum values). Allelic frequencies were determined by gene counting, and departures from the Hardy-Weinberg equilibrium (HWE) were investigated using chi-squared test. Linkage disequilibrium (LD) between the candidate polymorphism and the Thr92Ala polymorphism was examined using Lewontin's D′ |D′| and *r* ^2^ measures [34], [35]. Haplotypes constructed from the combination of these *DIO2* polymorphisms and their frequencies were inferred using the Phase 2.1 program, which uses a Bayesian statistical method [35], [36]. We also used this program to compare the distributions of *DIO2* haplotypes between patients with T2DM and nondiabetic subjects by permutation analyses of 1,000 random replicates [35].

Clinical and laboratory characteristics were compared using ANOVA, unpaired Student *t* test or chi-squared test, as appropriate. Variables with a skewed distribution were logarithmically transformed before analyses. To check if the candidate polymorphism was independently associated with T2DM, a multiple logistic regression analysis was performed with T2DM as the dependent variable and age, sex, and the possible informative polymorphism as independent variables. A linear regression analysis was performed to evaluate the independent association between the candidate polymorphism and IR parameters after adjusting for covariates (age, sex, BMI and use of T2DM medication). Haplotype interaction between the candidate polymorphism and the Thr92Ala polymorphism in modulating fasting insulin and HOMA-IR index was tested by general linear model univariate analyses (GLM), after adjusting for covariates (age, sex, BMI and use of T2DM medication). In these interaction analyses, each of the polymorphisms was modeled as a dichotomous variable.

All analyses were performed using the SPSS 18.0 (SPSS, Chicago, IL, USA). The level of statistical significance was set at P less than 0.05. Power calculations for the case-control study were done using the software PEPI, version 4.0, and showed that this study has a power of approximately 80% at a significance level of 0.05 to detect an OR of 1.35 or higher (for a 40% frequency of the minor allele).

## Results

### DIO2 screening and identification of candidate polymorphisms for association with insulin resistance

**Figure 1:**
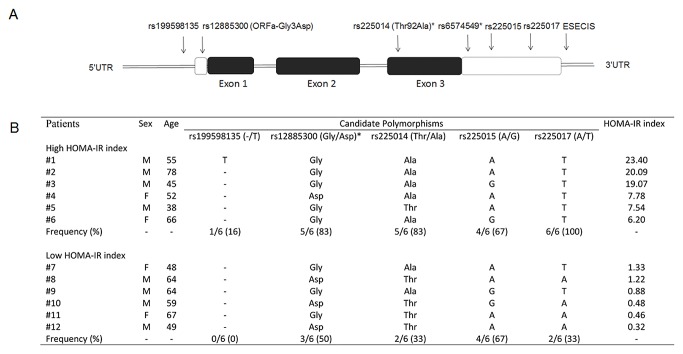
Candidate polymorphisms identified by sequencing of the *DIO2* gene. **A.** The vertical arrows show the five candidate variants in human *DIO2* gene identified through sequencing. Black boxes are coding regions. *polymorphisms associated with T2DM/IR/fasting insulin. ESECIS, Selenocysteine Insertion Sequence Element; 3′UTR = 3′-untranslated region. **B.** The characteristics of the 12 patients with T2DM selected for the screening of the *DIO2* gene. These patients had extreme HOMA-IR indexes and were selected from two subgroups according low or high HOMA-IR values.

An interim study comprising samples of 12 patients with T2DM was used to search for polymorphisms in the *DIO2* gene. This first phase study identified 448 amplicons (data not shown). Although no new mutation was detected in the data set, 5 previously described SNPs were identified (**Figure 1**). These SNPs included two polymorphisms in the 5′ flanking region (rs199598135, rs12885300), one polymorphism in exon 3 (rs225014 - Thr92Ala), and two in the 3′UTR (rs225015, rs225017).

The characteristics of the 12 patients with T2DM selected for the screening of the *DIO2* gene, as well as the occurrence of the 5 *DIO2* polymorphisms identified in these patients are shown in **Figure 1**. We observed that the T/T genotype of the rs225017 (A/T) polymorphism was more frequent in patients with a high HOMA-IR index (6 in 6) than in patients with a low HOMA-IR index (2 in 6). Moreover, the relative frequency of this polymorphism for white subjects in GenBank was 0.36 whereas in our sample was 0.66 (P = 0.129). This polymorphism also fills the criteria for selecting an informative polymorphism (see Material and Methods Section) and was then selected for further analysis in an association study. The Thr92Ala polymorphism also fulfilled the above criteria; however, this polymorphism was already reported in our sample population as being associated with both T2DM risk [27] and increased IR and insulin levels [37]. Thus, in the present study, we aimed to evaluate whether haplotypes constructed from the combination of Thr92Ala polymorphism with the rs225017 polymorphism are associated with IR in T2DM patients.

### The rs225017 variant and T2DM development in a case-control study

The baseline characteristics of the 1077 patients with T2DM and the 516 nondiabetic subjects included in the study were as follows: mean age was 59.2±10.1 years *vs*. 46.2±10.3 years (P<0.001), and males comprised 47% and 63% of the sample (P<0.001).

The T allele frequency of the rs225017 polymorphism did not differ significantly between patients with T2DM and nondiabetic subjects (0.51 *vs*. 0.48, P = 0.196). In addition, genotypes frequencies of this polymorphism were similar between T2DM patients and nondiabetic subjects (25.9% A/A, 47.2% A/T, 26.9% T/T vs. 28.5% A/A, 46.9% A/T, 24.6% T/T; P = 0.452). These findings did not change after logistic regression analysis adjusting for age and sex (OR = 1.15, 95% CI = 0.86–1.55; P = 0.354). Genotype frequencies of the rs225017 polymorphism were in HWE in patients with T2DM and nondiabetic subjects (P>0.05).

### The rs225017 DIO2 polymorphism is associated with insulin resistance in patients with T2DM

**Table 1** summarizes the clinical and laboratory characteristics of patients with T2DM grouped according to the different genotypes of the rs225017 polymorphism. Assuming a recessive model of inheritance, patients with A/A or A/T genotypes were grouped and compared with patients carrying the T/T genotype. T2DM duration (P = 0.009), sex proportion (P = 0.038) and use of metformin alone (P = 0.027) or metformin + sulfonylurea (P = 0.015) for treatment of T2DM were differentially distributed between patients with the T/T genotype and A allele carriers. It is worth mentioning that none of the variables showed in **Table 1** attained statistical significance when assuming dominant (A/A *vs*. A/T-T/T) or additive (A/A *vs*. T/T) models of inheritance (data not shown).

**Table 1:** Clinical and laboratory characteristics of patients with T2DM broken down according to *DIO2* rs225017 (T/A) polymorphism.

		Genotypes		
	Total (n = 1077)	A/A or A/T (n = 798)	T/T (n = 279)	P
Age (Years)	59.2±10.1	59.6±9.8	58.2±10.3	0.051
Duration of diabetes (Years)	11.7±8.8	12.1±9.0	10.5±7.9	0.009
Sex (% males)	47.0	49.0	42.0	0.038
BMI (kg/m^2^)	28.9±4.9	28.8±4.8	29.3±5.3	0.117
Total cholesterol (mmol/l)	5.4±1.2	5.4±1.2	5.5±1.2	0.419
Systolic BP (mm Hg)	141.9±23.2	141.9±23.7	141.8±21.8	0.953
Diastolic BP (mm Hg)	85.0±12.8	84.8±12.7	85.6±13.1	0.823
HDL cholesterol (mmol/l)	1.17±0.3	1.17±0.3	1.16±0,3	0.753
LDL cholesterol (mmol/l)	1.29±1.1	1.29±1.1	1.31±1.1	0.533
Serum creatinine (µmol/l)^a^	102.8 (35.4–937)	81.4 (26.5–937)	79.6 (35.4–540)	0.127
Triglycerides (mmol/l)^a^	2.00 (0.3–13.2)	2.00 (0.3–13.2)	1.99 (0.5–8.7)	0.821
Fasting plasma glucose (mmol/l)	9.4 (2.9–21.9)	9.3 (2.9–21.9)	9.8 (3.3–20.5)	0.144
SU/Met/SU + Met (%)	32.5/38.5/16.5	31.1/36.0/14.3	34.8/45.9/22.9	0.249/0.027/0.015
A1C (%)	7.2±2.0	7.1±2.0	7.3±1.8	0.343
Fasting insulin (UI/ml)^a^^,^ b	13.9 (1–46.1)	10.6 (1–44.9)	15.7 (3.2–46.1)	0.005
HOMA–IR index^a^^,^ b	3.8 (0.3–20.1)	3.5 (0.3–19.5)	5.2 (0.6–20.1)	0.005

Data are mean ± SD, median (minimum - maximum values) or %. A1C, glycated haemoglobin; BMI, body mass index; HOMA-IR, homeostasis model assessment - insulin resistance; Met, metformin; SU, sulfonylureas; WHR, waist-to-hip ratio; P values were estimated by χ^2^ or ANOVA, as appropriate.

a Variables which were logarithmically transformed before analyses.

b For comparisons of fasting insulin levels and HOMA-IR index among rs225017 genotypes, we analyzed only 227 individuals (162 individuals harboring the A/A or A/T genotypes and 65 individuals harboring the T/T genotype).

A subgroup of 227 patients with T2DM who were not receiving insulin was further analyzed for IR measurements. This subgroup was representative of the whole sample: mean age was 59.3±9.3 years (P = 0.803 for the comparison with the whole sample), mean T2DM duration was 11.2±8.2 years (P = 0.580), mean A1C was 7.3±1.7% (P = 0.12), and mean BMI was 28.9±4.7 kg/m^2^ (P = 0.89). Males comprised 45.3% (n = 103) of the sample (P = 0.45).

In this subgroup of patients, median fasting plasma insulin levels were higher in patients with the T/T genotype than in patients carrying the A allele [15.71 (3.2–46.1) *vs*. 10.57 (1–44.9), P = 0.005], whereas fasting plasma glucose levels did not differ significantly between groups (P = 0.144). Moreover, T/T genotype patients showed a higher HOMA-IR index than patients carrying the A allele [5.20 (0.6–20.1) *vs*. 3.50 (0.3–19.5), P = 0.005]. Because insulin sensitivity is known to be affected by multiple independent factors, multiple linear regression analyses were performed with HOMA-IR index (log_10_ HOMA-IR) or insulin level (log_10_ fasting insulin) as dependent variables. The T/T genotype of the rs225017 polymorphism remained significantly associated with HOMA-IR [standardized coefficient B for T/T genotype  = −0.366, 95% CI (−0.654–−0.078); P = 0.013] and insulin levels [standardized coefficient B for T/T genotype  = −0.268, 95% CI (−0.441–−0.096); P = 0.002], after adjusting for age, sex, BMI and use of medication for T2DM (mainly metformin or sulfonylureas).

### Association of the DIO2 rs225017 and Thr92Ala variants with insulin resistance

We used a Bayesian statistical method to estimate the frequencies of different haplotypes produced by the combination of the rs225017 and Thr92Ala polymorphisms in patients with T2DM. The first letter of the haplotypes refers to the Thr92Ala polymorphism, and the second, to the rs225017 polymorphism. The wild type haplotype (Thr/A) was observed in 49.0% of the sample, the Thr/T haplotype was observed in 18.0%, the Ala/A haplotype in 4.0%, and the Ala/T haplotype (double mutated) in 29.0% of the sample.

**Figure 2:**
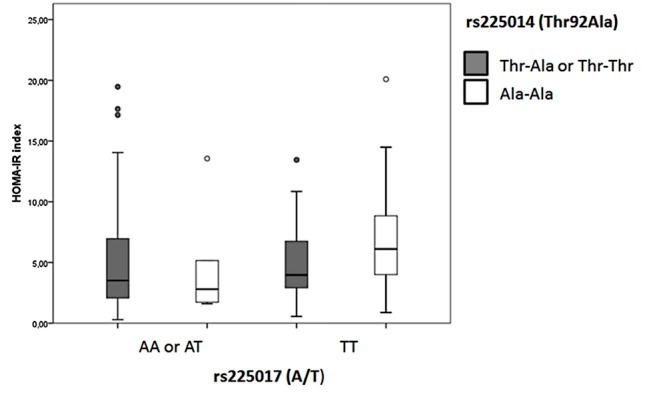
Possible interaction between rs225017 and Thr92Ala variants in modulation of insulin resistance. HOMA-IR indexes in T2DM patients according to different genotypes of *DIO2* rs225017 (A/T) and rs225014 (Thr92Ala) polymorphisms. Results are expressed as median (percentiles 25% and 75%). Circles represent outlier values. Interaction P value  = 0.010, adjusted for age, sex, BMI, and use of medication for T2DM.

Taking these results into account, we tested whether any haplotype constituted by the rs225017 and Thr92Ala polymorphisms might affect fasting plasma insulin levels or HOMA-IR index differently as compared with the effect of each polymorphism analyzed separately. To test this hypothesis, we performed GLM analyses using fasting insulin levels or the HOMA-IR index as the dependent variable, and age, sex, BMI, use of medication for T2DM and the polymorphic combinations of the two *DIO2* variants as independent variables. These interaction analyses are shown in **Table 2**. Patients carrying the Ala/Ala – T/T haplotype showed higher fasting insulin values than patients with other genotype combinations, adjusting for covariables (F = 11.072, P = 0.001). Likewise, patients carrying the Ala/Ala – T/T haplotype showed a higher HOMA-IR index than patients with other genotype combinations (F = 4.740; P = 0.010) (**Figure 2**).

**Table 2:** Interaction analyses between the *DIO2* rs225017 (T/A) and rs225014 (Thr92Ala) polymorphisms according to fasting plasma insulin and HOMA-IR index.

	rs225017						
	A/X			T/T			
Thr92Ala	Thr/X (n = 138)	Ala/Ala (n = 14)	P^a^	Thr/X (n = 35)	Ala/Ala (n = 30)	P^a^	F; P^b^
Age (years)	59.7±9.9	57.2±10.5	0.177	58.2±10.6	58.1±10.0	0.941	-
Sex (% males)	49.0	42.0	0.439	39.0	49.0	0.417	-
BMI (kg/m^2^)	28.8±4.9	28.6±4.5	0.865	29.4±5.5	29.3±5.1	0.959	-
SU/Met/SU + Met (%)	31.3/35.6/14.0	28.6/50.0/14.0	0.830/0.270/0.997	35.3/51.5/22.0	34.3/40.3/24.0	0.907/0.196/0.803	-
Fasting insulin (UI/ml)^c^^,^ d	10.8 (1–44.9)	8.6 (4.0–39.0)	0.794	11.9 (4.5–44.6)	16.8 (3.2–46.1)	0.011	11.072; 0.001
HOMA-IR index^c^^,^ e	3.5 (0.3–19.5)	2.8 (1.6–13.6)	0.916	3.9 (0.6–13.5)	6.1 (0.9–20.1)	0.034	4.740; 0.010

Data are mean ± SD, median (minimum - maximum values) or %. BMI, body mass index; HOMA-IR, homeostasis model assessment - insulin resistance; Met, metformin; SU, sulfonylureas.

a Data were analyzed using the Student *t* test or χ^2^, as appropriate.

b F and P values obtained from the general linear model-interaction analyses, after adjusting for age, sex, BMI and use of medication for T2DM.

c Variables which were logarithmically transformed before analyses.

d Adjusted R squared for fasting plasma insulin  = 0.135.

e Adjusted R squared for HOMA-IR  = 0.111.

To determine whether the rs225017 (A/T) and rs225014 (Thr92Ala) polymorphisms were in LD in T2DM patients, we used Lewontin's |D′| and *r* ^2^ measures. The analysis showed that the rs225017 polymorphism was in partial LD with the rs225014 polymorphism (|D′| = 0.811; *r* ^2^ = 0.365). These results were confirmed in the HAPMAP Project (http://hapmap.ncbi.nlm.nih.gov/).

## Discussion

Insulin resistance is a heterogeneous disorder influenced by multiple environmental and genetic factors [38], [39], [40]. Previous studies indicate that the Thr92Ala polymorphism in the *DIO2* gene is involved in IR pathogenesis [7], [23], [37]. Even though the Ala/Ala genotype was associated with lower D2 activity in thyroid biopsy samples, *in vitro* studies did not show any change in the biochemical properties of the mutated enzyme, suggesting that this SNP might be only a polymorphic marker [7]. Therefore, here we searched for other informative SNPs in the *DIO2* gene which might be associated with IR or T2DM. We report a new *DIO2* polymorphism associated with increased IR in T2DM patients. Homozygosis for the allele T of the rs225017 (A/T) polymorphism was associated with IR in patients with T2DM, whereas patients carrying a combination of the T/T genotype of the rs225017 polymorphism and the Ala/Ala genotype of the Thr92Ala polymorphism showed an increased HOMA-IR index as compared with patients with other genotype combinations, suggesting that they might interact in the modulation of IR.

The rs205017 (A/T) polymorphism is located at the 3′UTR of the *DIO2* gene. In selenoenzymes, the 3′UTR contains the selenocysteine insertion sequence (SECIS), which is very important for looping formation in the mechanism of cis-acting mRNA structure of selenocysteine insertion [41], [42]. The 3′UTR is also the main site for ligation of microRNAs (miRNAs) that can function as translational repressors or triggers of transcript degradation by partially pairing to this region in target mRNAs [43]. Currently, it is universally recognized that miRNAs are major regulators of gene expression and key controllers of several biologic and pathologic processes, as such those involved in the T2DM pathogenesis [44]. Hence, it is reasonable to speculate that the rs205017 polymorphism could be involved in a ligation site for a given miRNA, decreasing the *DIO2* expression. Therefore, we used the TargetScan software to look for potential miRNA targets in the *DIO2* gene [45]. Six predicted interaction regions with miRNA families were found in the *DIO2* sequence: miR-9, miR-29abcd, miR-30abcdef, miR-193, miR-203 and miR-216a. Interestingly, miR-9, miR-29 and miR-30 seem to have a role in insulin resistance [44], [46]. Although the rs225017 SNP is not located at any of these miRNA regions, it is close to some of them and, therefore, we could not rule out a potential effect of this SNP on *DIO2* gene expression. Alternatively, it could be obstructing the mechanism of selenocysteine insertion and, thereby, reducing the amount of D2 available for the activation of T3. In this context, polymorphic D2, generated from the Ala/T haplotype sequence, might generate less T3 in skeletal muscle and adipocytes, which, consequently, may create a local state of intracellular hypothyroidism, decreasing the expression of genes involved in metabolism, such as GLUT4, and leading to IR.

Recently, the *DIO2* rs6574549 polymorphism, also located at the 3′UTR, was associated with elevated fasting insulin levels and higher insulin action, but not with T2DM in Pima Indians [47]. This polymorphism may also be a candidate for the functional variant associated with IR. The rs6574549 polymorphism is in strong LD with the Thr92Ala [47] and rs205017 [48] polymorphisms (LD>0.8; HAPMAP Project, http://hapmap.ncbi.nlm.nih.gov/). Therefore, further studies are necessary to determine: 1) which polymorphism is the functional variant interacting with the Thr92Ala polymorphism in the modulation of IR; 2) whether there is another functional polymorphism in the *DIO2* gene yet to be identified; and 3) whether these polymorphisms have combined effects on D2 function. The results presented here add to the understanding of the molecular interactions controlling IR and might partially explain the association with the Thr92Ala polymorphism.

Some factors unrelated to the rs205017 and Thr92Ala polymorphisms may have interfered with the findings of the present study. First, medications for T2DM treatment may have played a role because some are known to affect insulin sensitivity. However, we minimized such possibility by excluding insulin-treated patients from the group of 227 patients analyzed for IR. Furthermore, the rs205017 variant and the Ala/Ala-T/T haplotype remained independently associated with the HOMA-IR index and plasma insulin levels after adjusting for medications for T2DM, including metformin and sulfonylurea. In addition, none of the patients were using thiazolidinediones, agents that change insulin sensitivity. Second, IR was assessed by calculation of the HOMA-IR index rather than by the reference method, the euglycaemic-hyperinsulinemic clamp [32], [49], [50]. Although the HOMA-IR index is only an approximate estimate of IR, it is simple to calculate and shows a good correlation with the reference method [32], [49]; therefore, it is a good approach for cohort and epidemiological studies [50]. Third, we cannot rule out the possibility of stratification bias in our sample, although we studied only self-defined white subjects and thus reduced the risk of false positive or false negative associations due to this bias. In addition, the 227 patients with T2DM analyzed for IR were representative of the whole sample (n = 1077) with respect to age, T2DM duration, A1c, sex, BMI and presence of arterial hypertension. The frequencies of the rs205017 and Thr92Ala polymorphisms were also similar between these 227 patients and the whole T2DM sample (data not shown). Fourth, screening only 12 patients with T2DM for the occurrence of *DIO2* polymorphisms might have failed to detect rare polymorphisms [49]; however, this number was enough to detect most representative variants, which are sufficiently polymorphic to warrant association studies. Finally, our results might represent a type 1 error, but the scientific plausibility of the reported association provides evidence against it.

In summary, the *DIO2* rs225017 (A/T) polymorphism is a new polymorphism associated with IR in white patients with T2DM, and it seems to interact with the Thr92Ala polymorphism in the modulation of this characteristic. Further studies are needed to evaluate the functional and epidemiological importance of the rs205017 polymorphism in IR and T2DM.

## Supporting Information

Table S1 **Primer and probe sequences used for sequencing analyses of the** ***DIO2*** **gene or genotyping of** ***DIO2*** **polymorphisms.**
(DOC)

## References

[1] Yen PM (2001) Physiological and molecular basis of thyroid hormone action. Physiol Rev 81: 1097–1142. 11427693 10.1152/physrev.2001.81.3.1097

[2] Maia AL, Goemann IM, Meyer EL, Wajner SM (2011) Deiodinases: the balance of thyroid hormone: type 1 iodothyronine deiodinase in human physiology and disease. J Endocrinol 209: 283–297. 21415143 10.1530/JOE-10-0481

[3] Maia AL, Kim BW, Huang SA, Harney JW, Larsen PR (2005) Type 2 iodothyronine deiodinase is the major source of plasma T3 in euthyroid humans. J Clin Invest 115: 2524–2533. 16127464 10.1172/JCI25083 PMC1190373

[4] Croteau W, Davey JC, Galton VA, St Germain DL (1996) Cloning of the mammalian type II iodothyronine deiodinase. A selenoprotein differentially expressed and regulated in human and rat brain and other tissues. J Clin Invest 98: 405–417. 8755651 10.1172/JCI118806 PMC507444

[5] Itagaki Y, Yoshida K, Ikeda H, Kaise K, Kaise N, et al (1990) Thyroxine 5′-deiodinase in human anterior pituitary tumors. J Clin Endocrinol Metab 71: 340–344. 2380333 10.1210/jcem-71-2-340

[6] Salvatore D, Tu H, Harney JW, Larsen PR (1996) Type 2 iodothyronine deiodinase is highly expressed in human thyroid. J Clin Invest 98: 962–968. 8770868 10.1172/JCI118880 PMC507511

[7] Canani LH, Capp C, Dora JM, Meyer EL, Wagner MS, et al (2005) The type 2 deiodinase A/G (Thr92Ala) polymorphism is associated with decreased enzyme velocity and increased insulin resistance in patients with type 2 diabetes mellitus. J Clin Endocrinol Metab 90: 3472–3478. 15797963 10.1210/jc.2004-1977

[8] Grozovsky R, Ribich S, Rosene ML, Mulcahey MA, Huang SA, et al (2009) Type 2 deiodinase expression is induced by peroxisomal proliferator-activated receptor-gamma agonists in skeletal myocytes. Endocrinology 150: 1976–1983. 19036883 10.1210/en.2008-0938 PMC2659265

[9] Heemstra KA, Soeters MR, Fliers E, Serlie MJ, Burggraaf J, et al (2009) Type 2 iodothyronine deiodinase in skeletal muscle: effects of hypothyroidism and fasting. J Clin Endocrinol Metab 94: 2144–2150. 19293265 10.1210/jc.2008-2520

[10] Steinsapir J, Bianco AC, Buettner C, Harney J, Larsen PR (2000) Substrate-induced down-regulation of human type 2 deiodinase (hD2) is mediated through proteasomal degradation and requires interaction with the enzyme's active center. Endocrinology 141: 1127–1135. 10698189 10.1210/endo.141.3.7355

[11] Gereben B, Goncalves C, Harney JW, Larsen PR, Bianco AC (2000) Selective proteolysis of human type 2 deiodinase: a novel ubiquitin-proteasomal mediated mechanism for regulation of hormone activation. Mol Endocrinol 14: 1697–1708. 11075806 10.1210/mend.14.11.0558

[12] Frojdo S, Vidal H, Pirola L (2009) Alterations of insulin signaling in type 2 diabetes: a review of the current evidence from humans. Biochim Biophys Acta 1792: 83–92. 19041393 10.1016/j.bbadis.2008.10.019

[13] Chidakel A, Mentuccia D, Celi FS (2005) Peripheral metabolism of thyroid hormone and glucose homeostasis. Thyroid 15: 899–903. 16131332 10.1089/thy.2005.15.899

[14] Kim SR, Tull ES, Talbott EO, Vogt MT, Kuller LH (2002) A hypothesis of synergism: the interrelationship of T3 and insulin to disturbances in metabolic homeostasis. Med Hypotheses 59: 660–666. 12445506 10.1016/s0306-9877(02)00211-6

[15] Weinstein SP, O'Boyle E, Haber RS (1994) Thyroid hormone increases basal and insulin-stimulated glucose transport in skeletal muscle. The role of GLUT4 glucose transporter expression. Diabetes 43: 1185–1189. 7926286 10.2337/diab.43.10.1185

[16] Torrance CJ, Devente JE, Jones JP, Dohm GL (1997) Effects of thyroid hormone on GLUT4 glucose transporter gene expression and NIDDM in rats. Endocrinology 138: 1204–1214. 9048628 10.1210/endo.138.3.4981

[17] Dimitriadis G, Baker B, Marsh H, Mandarino L, Rizza R, et al (1985) Effect of thyroid hormone excess on action, secretion, and metabolism of insulin in humans. Am J Physiol 248: E593–601. 3887944 10.1152/ajpendo.1985.248.5.E593

[18] Dimitriadis G, Maratou E, Alevizaki M, Boutati E, Psara K, et al (2005) Thyroid hormone excess increases basal and insulin-stimulated recruitment of GLUT3 glucose transporters on cell surface. Horm Metab Res 37: 15–20. 15702433 10.1055/s-2005-861026

[19] Rochon C, Tauveron I, Dejax C, Benoit P, Capitan P, et al (2003) Response of glucose disposal to hyperinsulinaemia in human hypothyroidism and hyperthyroidism. Clin Sci (Lond) 104: 7–15. 12519082 10.1042/

[20] Dubaniewicz A, Kaciuba-Uscilko H, Nazar K, Budohoski L (1989) Sensitivity of the soleus muscle to insulin in resting and exercising rats with experimental hypo- and hyper-thyroidism. Biochem J 263: 243–247. 2690814 10.1042/bj2630243 PMC1133414

[21] Marsili A, Aguayo-Mazzucato C, Chen T, Kumar A, Chung M, et al (2011) Mice with a targeted deletion of the type 2 deiodinase are insulin resistant and susceptible to diet induced obesity. PLoS One 6: e20832. 21698184 10.1371/journal.pone.0020832 PMC3116839

[22] Coppotelli G, Summers A, Chidakel A, Ross JM, Celi FS (2006) Functional characterization of the 258 A/G (D2-ORFa-Gly3Asp) human type-2 deiodinase polymorphism: a naturally occurring variant increases the enzymatic activity by removing a putative repressor site in the 5′ UTR of the gene. Thyroid 16: 625–632. 16889485 10.1089/thy.2006.16.625

[23] Mentuccia D, Proietti-Pannunzi L, Tanner K, Bacci V, Pollin TI, et al (2002) Association between a novel variant of the human type 2 deiodinase gene Thr92Ala and insulin resistance: evidence of interaction with the Trp64Arg variant of the beta-3-adrenergic receptor. Diabetes 51: 880–883. 11872697 10.2337/diabetes.51.3.880

[24] Maia AL, Dupuis J, Manning A, Liu C, Meigs JB, et al (2007) The type 2 deiodinase (DIO2) A/G polymorphism is not associated with glycemic traits: the Framingham Heart Study. Thyroid 17: 199–202. 17381351 10.1089/thy.2006.0298

[25] Mentuccia D, Thomas MJ, Coppotelli G, Reinhart LJ, Mitchell BD, et al (2005) The Thr92Ala deiodinase type 2 (DIO2) variant is not associated with type 2 diabetes or indices of insulin resistance in the old order of Amish. Thyroid 15: 1223–1227. 16356084 10.1089/thy.2005.15.1223

[26] Grarup N, Andersen MK, Andreasen CH, Albrechtsen A, Borch-Johnsen K, et al (2007) Studies of the common DIO2 Thr92Ala polymorphism and metabolic phenotypes in 7342 Danish white subjects. J Clin Endocrinol Metab 92: 363–366. 17077128 10.1210/jc.2006-1958

[27] Dora JM, Machado WE, Rheinheimer J, Crispim D, Maia AL (2010) Association of the type 2 deiodinase Thr92Ala polymorphism with type 2 diabetes: case-control study and meta-analysis. Eur J Endocrinol 163: 427–434. 20566590 10.1530/EJE-10-0419

[28] Peeters RP, van Toor H, Klootwijk W, de Rijke YB, Kuiper GG, et al (2003) Polymorphisms in thyroid hormone pathway genes are associated with plasma TSH and iodothyronine levels in healthy subjects. J Clin Endocrinol Metab 88: 2880–2888. 12788902 10.1210/jc.2002-021592

[29] Adams DR, Sincan M, Fuentes Fajardo K, Mullikin JC, Pierson TM, et al (2011) Analysis of DNA sequence variants detected by high-throughput sequencing. Hum Mutat 33: 599–608. 10.1002/humu.22035 PMC3959770 22290882

[30] Cooper DN, Chen JM, Ball EV, Howells K, Mort M, et al (2010) Genes, mutations, and human inherited disease at the dawn of the age of personalized genomics. Hum Mutat 31: 631–655. 20506564 10.1002/humu.21260

[31] Canani LH, Costa LA, Crispim D, Goncalves Dos Santos K, Roisenberg I, et al (2005) The presence of allele D of angiotensin-converting enzyme polymorphism is associated with diabetic nephropathy in patients with less than 10 years duration of Type 2 diabetes. Diabet Med 22: 1167–1172. 16108844 10.1111/j.1464-5491.2005.01622.x

[32] Bonora E, Targher G, Alberiche M, Bonadonna RC, Saggiani F, et al (2000) Homeostasis model assessment closely mirrors the glucose clamp technique in the assessment of insulin sensitivity: studies in subjects with various degrees of glucose tolerance and insulin sensitivity. Diabetes Care 23: 57–63. 10857969 10.2337/diacare.23.1.57

[33] Seligman BG, Biolo A, Polanczyk CA, Gross JL, Clausell N (2000) Increased plasma levels of endothelin 1 and von Willebrand factor in patients with type 2 diabetes and dyslipidemia. Diabetes Care 23: 1395–1400. 10977040 10.2337/diacare.23.9.1395

[34] Hedrick PW (1987) Gametic disequilibrium measures: proceed with caution. Genetics 117: 331–341. 3666445 10.1093/genetics/117.2.331 PMC1203208

[35] Barrett JC, Fry B, Maller J, Daly MJ (2005) Haploview: analysis and visualization of LD and haplotype maps. Bioinformatics 21: 263–265. 15297300 10.1093/bioinformatics/bth457

[36] Stephens M, Smith NJ, Donnelly P (2001) A new statistical method for haplotype reconstruction from population data. Am J Hum Genet 68: 978–989. 11254454 10.1086/319501 PMC1275651

[37] Estivalet AA, Leiria LB, Dora JM, Rheinheimer J, Boucas AP, et al (2010) D2 Thr92Ala and PPARgamma2 Pro12Ala polymorphisms interact in the modulation of insulin resistance in type 2 diabetic patients. Obesity (Silver Spring) 19: 825–832. 20930717 10.1038/oby.2010.231

[38] Irvin MR, Wineinger NE, Rice TK, Pajewski NM, Kabagambe EK, et al (2011) Genome-wide detection of allele specific copy number variation associated with insulin resistance in African Americans from the HyperGEN study. PLoS One 6: e24052. 21901158 10.1371/journal.pone.0024052 PMC3162025

[39] North KE, Williams K, Williams JT, Best LG, Lee ET, et al (2003) Evidence for genetic factors underlying the insulin resistance syndrome in american indians. Obes Res 11: 1444–1448. 14694207 10.1038/oby.2003.193

[40] North KE, Almasy L, Goring HH, Cole SA, Diego VP, et al (2005) Linkage analysis of factors underlying insulin resistance: Strong Heart Family Study. Obes Res 13: 1877–1884. 16339117 10.1038/oby.2005.230

[41] Mix H, Lobanov AV, Gladyshev VN (2007) SECIS elements in the coding regions of selenoprotein transcripts are functional in higher eukaryotes. Nucleic Acids Res 35: 414–423. 17169995 10.1093/nar/gkl1060 PMC1802603

[42] Ryan K, Bauer DL (2008) Finishing touches: post-translational modification of protein factors involved in mammalian pre-mRNA 3′ end formation. Int J Biochem Cell Biol 40: 2384–2396. 18468939 10.1016/j.biocel.2008.03.016 PMC2548416

[43] Ambros V (2004) The functions of animal microRNAs. Nature 431: 350–355. 15372042 10.1038/nature02871

[44] Guay C, Regazzi R (2013) Circulating microRNAs as novel biomarkers for diabetes mellitus. Nat Rev Endocrinol 9: 513–521. 23629540 10.1038/nrendo.2013.86

[45] Lewis BP, Burge CB, Bartel DP (2005) Conserved seed pairing, often flanked by adenosines, indicates that thousands of human genes are microRNA targets. Cell 120: 15–20. 15652477 10.1016/j.cell.2004.12.035

[46] Guay C, Roggli E, Nesca V, Jacovetti C, Regazzi R (2011) Diabetes mellitus, a microRNA-related disease? Transl Res 157: 253–264. 21420036 10.1016/j.trsl.2011.01.009

[47] Nair S, Muller YL, Ortega E, Kobes S, Bogardus C, et al (2012) Association analyses of variants in the DIO2 gene with early-onset type 2 diabetes mellitus in Pima Indians. Thyroid 22: 80–87. 22142372 10.1089/thy.2010.0455 PMC3247704

[48] Altshuler DM, Gibbs RA, Peltonen L, Altshuler DM, Gibbs RA, et al (2010) Integrating common and rare genetic variation in diverse human populations. Nature 467: 52–58. 20811451 10.1038/nature09298 PMC3173859

[49] Emoto M, Nishizawa Y, Maekawa K, Hiura Y, Kanda H, et al (1999) Homeostasis model assessment as a clinical index of insulin resistance in type 2 diabetic patients treated with sulfonylureas. Diabetes Care 22: 818–822. 10332688 10.2337/diacare.22.5.818

[50] Wallace TM, Levy JC, Matthews DR (2004) Use and abuse of HOMA modeling. Diabetes Care 27: 1487–1495. 15161807 10.2337/diacare.27.6.1487

