# Supplementary material for: The rs225017 Polymorphism in the 3′UTR of the Human DIO2 Gene Is Associated with Increased Insulin Resistance
Source: PLoS One. 2014 Aug 8;9(8):e103960. doi: 10.1371/journal.pone.0103960 (PMC4126657; doi:10.1371/journal.pone.0103960)
Supplement: Table S1 — Primer and probe sequences used for sequencing analyses of the DIO2 gene or genotyping of DIO2 polymorphisms. (DOC) [file pone.0103960.s001.doc]

**Table S1**. Primer and probe sequences used for sequencing analyses of the *DIO2* gene or genotyping of *DIO2* polymorphisms

|  | Sequences | Site | Size |
| --- | --- | --- | --- |
| Sequencing analyses ^a^ |  |  |  |
| #1 | F 5'- TAGGTCACAGATCTTACAAAG - 3' / R 5'- CAGTGAGTCTCCCTGACGCCT - 3' | -1000 to -500 | 500 bp |
| #2 | F 5'- GGCTGCAGAGAGAGGCACTT - 3' / R 5'- TGGAGTGTGCCCATCAATTC - 3' | -500 to +1 | 500 bp |
| #3 | F 5'- CCCCACCCCCTTTATCACCA - 3' / R 5'- GAGGTCAAGTGGCTGAGCCA - 3' | +1 to 491 | 491 bp |
| #4 | F 5'- GCTTGGAAATGAAAGTAGAA - 3' / R 5'- GAATGACCGAGTCATAGAGA - 3' | 301 to 807 | 506 bp |
| #5 | F 5'- CACAAGGGAACTGACTCAGG - 3' / R 5'- | 680 to 1197 | 517 bp |
|  | ACTCCCAAATCACAGCAAGA - 3' |  |  |
| #6 | F 5'- TTACGGGGTAGCCTTTGAAC - 3' / R 5'- TTTTCTTCTGGTCTCAAAGC - 3' | 800 to 1289 | 489 bp |
| #7 | F 5'- TCCAAGTCCACTCGCGGAGA - 3' / R 5'- CCTCAGCCTCCCATCAAGCA - 3' | 840 to 1325 | 485 bp |
| #8 | F 5'- TCGTGGGGAGAGCAAAGAAT - 3' / R 5'- ATTTTGTTGAGGAGCCAGGG - 3' | 5600 to 6097 | 497 bp |
| #9 | F 5'- TCGTGGGGAGAGCAAAGAAT - 3' / R 5'- ATTTTGTTGAGGAGCCAGGG - 3' | 6000 to 6500 | 500 bp |
| #10 | F 5'- TCTTCAGTGACTATACAGAATG - 3' / R 5'- AATGTAGACCAGCAGGAAGT - 3' | 8658 to 9155 | 497 bp |
| #11 | F 5'- CAAACTGGTGGAAGAGTTCT - 3' / R 5'- CCTGTCTTTCAGTAAGCCAA - 3' | 9104 to 9603 | 499 bp |
| #12 | F 5'- CCTATTGGCTTACTGAAAGA - 3' / R 5'- AATTTCTGGGGTATGAAGAC - 3' | 9580 to 10069 | 489 bp |
| #13 | F 5'- ATATTTGTAATTGTGAGGGG - 3' / R 5'- GTCAATGGAAATTCCATGAT - 3' | 10426 to 10921 | 495 bp |
| #14 | F 5'- CACATTCACACTGTTGTCCTT - 3' / R 5'- CCTCTACCTCAAAATAATGAGT - 3' | 11675 to 12170 | 495 bp |
| #15 | F 5'- CCTACTTTGTATAGCTAAGTGAC - 3' / R 5'- GAACAAATGTCCAGATTCAT - 3' | 12705 to 13184 | 479 bp |
| #16 | F 5'- TTGAAAACAAACTTCTCGCA - 3' / R 5'- CACATCCCCCAATCCTAATA - 3' | 13681 to 14179 | 498 bp |
| **Genotyping** ^b^ |  |  |  |
| rs225014 (Thr92Ala) | F 5'-GGTACCATTGCCACTGTTGTCA-3' / R 5'- GTCAGGTGAAATTGGGTGAGGAT-3'  FAM-5'- ATGTCTCCAGTGCAGAA-3' / VIC-5'-TTTGGTTCTTGCACACCTAGTTCT-3' | 984 | 100 bp |
| rs225017 (A/T) | F 5'- TTTGGTTCTTGCACACCTAGTTCT -3' /  R 5'- AAAATGGATAGAAAAAAACTAAAGTTGAAA ATACA 3'  FAM-5'- CACTCTTTTCTCATTTCAGA -3' / VIC-5'- CACTCTTTTCTCAATTCAGA -3' | 3338 | 100 bp |

F = forward primers and R = reverse primers. ^a^ For sequencing analysis, primers were designed using human *DIO2* gene (NM_000793.5) and Vector NTI® Software (Invitrogen). ^b^ For genotyping of polymorphisms, primers and probes were designed by Life Technologies (Custom TaqMan Genotyping Assays).
